# Characterization of Neurophysiologic and Neurocognitive Biomarkers for Use in Genomic and Clinical Outcome Studies of Schizophrenia

**DOI:** 10.1371/journal.pone.0039434

**Published:** 2012-07-03

**Authors:** Gregory A. Light, Neal R. Swerdlow, Anthony J. Rissling, Allen Radant, Catherine A. Sugar, Joyce Sprock, Marlena Pela, Mark A. Geyer, David L. Braff

**Affiliations:** 1 VISN-22 Mental Illness, Research, Education, and Clinical Center (MIRECC), San Diego VA Health Care System, La Jolla, California, United States of America; 2 Department of Psychiatry, University of California San Diego, La Jolla, California, United States of America; 3 Department of Psychiatry and Behavioral Sciences, University of Washington, Seattle, Washington, United States of America; 4 Departments of Psychiatry and Biostatistics, University of California Los Angeles, Los Angeles, California, United States of America; University of Minnesota, United States of America

## Abstract

**Background:**

Endophenotypes are quantitative, laboratory-based measures representing intermediate links in the pathways between genetic variation and the clinical expression of a disorder. Ideal endophenotypes exhibit deficits in patients, are stable over time and across shifts in psychopathology, and are suitable for repeat testing. Unfortunately, many leading candidate endophenotypes in schizophrenia have not been fully characterized simultaneously in large cohorts of patients and controls across these properties. The objectives of this study were to characterize the extent to which widely-used neurophysiological and neurocognitive endophenotypes are: 1) associated with schizophrenia, 2) stable over time, independent of state-related changes, and 3) free of potential practice/maturation or differential attrition effects in schizophrenia patients (SZ) and nonpsychiatric comparison subjects (NCS). Stability of clinical and functional measures was also assessed.

**Methods:**

Participants (SZ n = 341; NCS n = 205) completed a battery of neurophysiological (MMN, P3a, P50 and N100 indices, PPI, startle habituation, antisaccade), neurocognitive (WRAT-3 Reading, LNS-forward, LNS-reorder, WCST-64, CVLT-II). In addition, patients were rated on clinical symptom severity as well as functional capacity and status measures (GAF, UPSA, SOF). 223 subjects (SZ n = 163; NCS n = 58) returned for retesting after 1 year.

**Results:**

Most neurophysiological and neurocognitive measures exhibited medium-to-large deficits in schizophrenia, moderate-to-substantial stability across the retest interval, and were independent of fluctuations in clinical status. Clinical symptoms and functional measures also exhibited substantial stability. A Longitudinal Endophenotype Ranking System (LERS) was created to rank neurophysiological and neurocognitive biomarkers according to their effect sizes across endophenotype criteria.

**Conclusions:**

The majority of neurophysiological and neurocognitive measures exhibited deficits in patients, stability over a 1-year interval and did not demonstrate practice or time effects supporting their use as endophenotypes in neural substrate and genomic studies. These measures hold promise for informing the “gene-to-phene gap” in schizophrenia research.

## Introduction

One prominent strategy for deconstructing complex, heritable neuropsychiatric disorders such as schizophrenia is to examine discrete, genetically determined “endophenotypes” that are part of the illness and detected in the laboratory rather than by “the naked eye” of the clinical interview [1]. Endophenotypes may be useful for deconstructing the complexity of clinical, neural substrate, and genetic underpinnings of the disorder [2], [3]. Several criteria for viable endophenotypes have been proposed [1], [4]–[6]. While there is some variability in the criteria, in general, endophenotypes are a subset of biomarkers that: 1) are associated with the illness, i.e., exhibit deficits in patients; 2) are stable over time; 3) are relatively independent of fluctuations in clinical symptoms; 4) show similar, though often lesser deficits in clinically unaffected family members; and 5) are heritable. This study addresses criteria 1–3 above. Thus, endophenotypes are quantitative, laboratory-based measures that represent intermediate links in the pathways between genetic variation and the clinical expression of the disorder that can uniquely inform the “gene-to-phene” knowledge gap.

**Figure 1:**
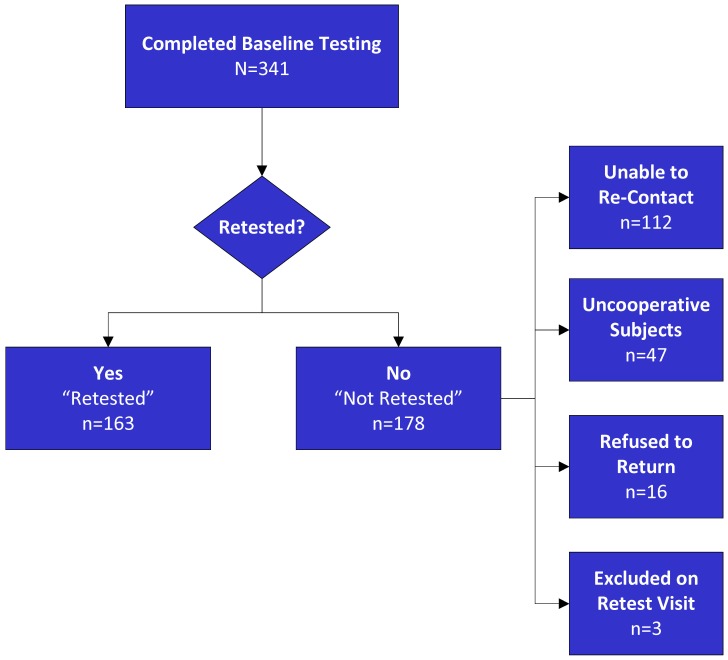
Schizophrenia patient study enrollment and reasons for not being retested.

Some widely used candidate neurophysiological endophenotypes in schizophrenia include prepulse inhibition of the acoustic startle reflex (PPI), P50 and N100 event-related potential amplitudes and gating, oculomotor antisaccade, mismatch negativity (MMN), and the P3a event-related potentials [2], [6], [7]. Commonly used neurocognitive endophenotypes include measures of attention, working memory, verbal recall, perseverative thinking and rule learning in response to verbal feedback [8], [9].

Increasingly, these and other neurophysiological and neurocognitive measures are used as biomarkers in clinical trials for “proof of concept” studies designed to determine whether a drug has a detectable “neurobiological signal” or as outcome measures to determine if a drug improves cognition [10]–[15]. In this context, the Measurement and Treatment Research to Improve Cognition in Schizophrenia (MATRICS) expert panel has established criteria that are considered “essential” for measure selection in clinical trial studies designed to improve cognition in schizophrenia [16]. These criteria are: 1) good test-retest reliability; 2) utility as a repeated measure (i.e., no practice effects); 3) a relationship to functional outcome; 4) a potential response to pharmacologic agents; and 5) practicality/tolerability. Thus, although endophenotypes and biomarkers share some common desirable characteristics [17], [18], further validation is required before treatment and clinical trial applications using these measures can be fully implemented.

**Figure 2:**
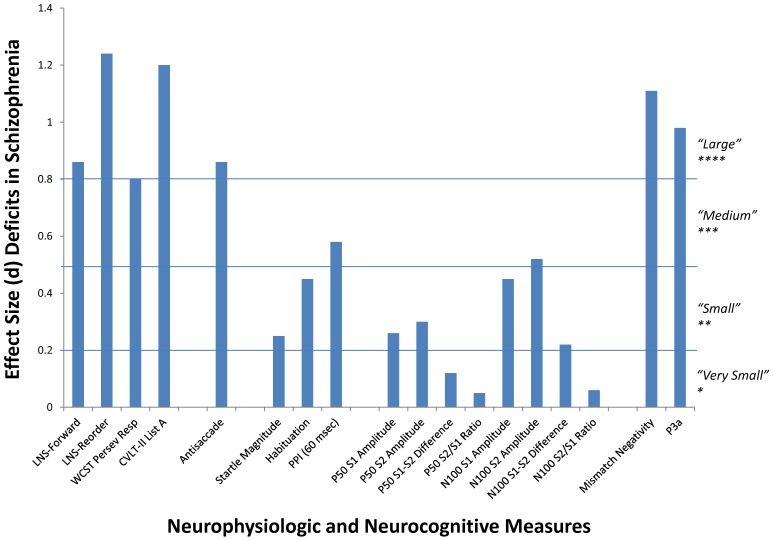
Deficits in schizophrenia patients across measures. Effect sizes (Cohen’s d) calculated from group main effects (Table 2) collapsed across time.

**Figure 3:**
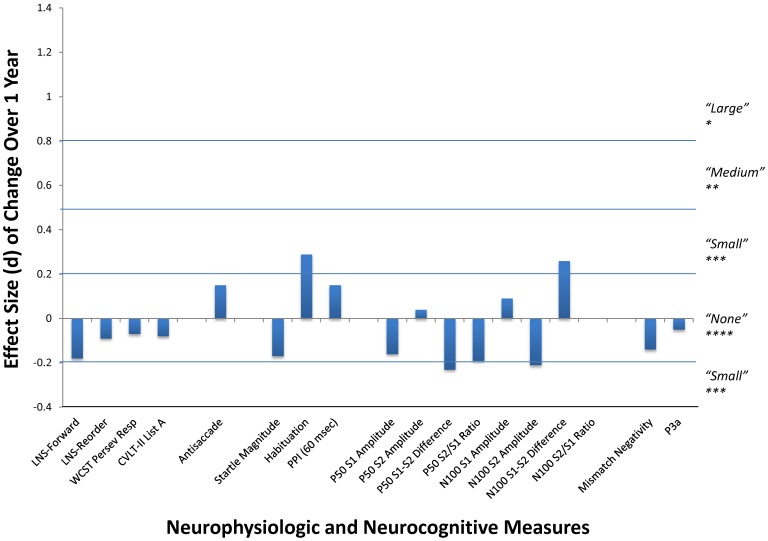
Changes in measures over 1 year retest interval in schizophrenia patients. Effect sizes (Cohen’s d) of changes in neurocognitive and neurophysiological measures across the retest interval.

The long-term stability of many neurocognitive measures in schizophrenia outpatients appears to be well-established (e.g., [19], [20]). Relatively few studies, however, have examined the test-retest reliability of commonly used neurophysiological biomarkers in schizophrenia patients [17], [21], [22]. Longitudinal studies of at least 6 months to one year are essential for disentangling state and trait influences and detecting enduring relationships among endophenotypes and clinical outcome measures (e.g., [23]–[25]). Thus, the aims of the present study were to characterize the extent to which a battery of 15 frequently used but not as yet fully validated candidate neurophysiological and neurocognitive measures fulfill many of the established criteria of endophenotypes and biomarkers of drug response including the extent to which measures are deficient in a large cohort of schizophrenia patients, stable over 1 year, and independent of symptom fluctuations in schizophrenia outpatients relative to nonpsychiatric comparison subjects (NCS). Secondary analyses also included assessments of potential practice effects (i.e., subjects’ performance improves due to increased familiarity with the test) and differential attrition (i.e., differences in baseline characteristics in patients who returned vs. failed to return for repeat testing).

**Table 1:** Assessment of differential attrition.

Schizophrenia Patients (N = 341)	Not-Retested (n = 178)		Retested (n = 163)		d
	Mean	SD	Mean	SD	
Demographic Characteristics					
Gender (% male)	71.91	–	73.00	–	–
Age	43.38	10.06	45.49	9.37	−0.22
Years of Education Completed	11.84	2.27	11.98	1.99	−0.07
Age of Illness Onset	21.59	7.36	21.88	7.19	−0.04
Duration of Illness	21.91	10.61	23.61	10.30	−0.16
Number of Hospitalizations	9.56	14.48	8.10	11.90	0.11
Hearing Threshold 1000 Hz	18.67	8.30	18.95	7.86	−0.03
Clinical and Functional Characteristics					
SAPS Total Score	9.39	3.94	8.32	4.38	0.26
SANS Total Score	13.89	3.95	13.60	4.55	0.07
Global Assessment of Functioning Scale	40.70	6.50	41.75	8.11	−0.14
Scale of Functioning Total Score	46.50	6.09	47.57	6.27	−0.17
UCSD Performance Based Skills Assessment (Total)	77.58	15.04	79.09	13.56	−0.11
Neurocognitive Performances					
Single-word reading (WRAT-3 Reading)	43.01	7.95	44.23	6.88	−0.16
Simple Attention (LNS-Forward)	11.34	3.05	11.90	3.04	−0.18
Working Memory (LNS-Reorder)	7.27	2.79	7.52	2.67	−0.09
Perseverative Thinking (WCST-64)	22.71	17.77	23.89	17.40	−0.07
Immediate Verbal Recall (CVLT-II Trials 1–5 Total)	34.41	10.49	35.33	11.92	−0.08
Neurophysiological Measures					
Antisaccade Proportion Correct	0.54	0.25	0.50	0.28	0.15
Startle Reactivity (Block 1 pulse alone magnitude)	72.29	56.22	81.70	53.70	−0.17
Startle Habituation	56.60	29.48	46.55	39.95	0.29
Prepulse Inhibition (30 msec)	34.69	26.34	29.28	23.70	0.22
Prepulse Inhibition (60 msec)	47.35	24.86	43.41	26.32	0.15
Prepulse Inhibition (120 msec)	62.57	25.12	58.23	27.49	0.17
P50 Amplitude (S1)	2.26	1.62	2.53	1.71	−0.16
P50 Amplitude (S2)	1.27	1.13	1.23	1.06	0.04
S1–S2 Difference	1.00	1.25	1.30	1.34	−0.23
P50 Suppression (%)	37.37	45.43	45.38	40.87	−0.19
N100 Amplitude (S1)	−2.53	2.23	−2.74	2.41	0.09
N100 Amplitude (S2)	−1.73	1.59	−1.43	1.23	−0.21
S1–S2 Difference	−0.78	1.85	−1.31	2.20	0.26
N100 Suppression (%)	–	–	–	–	–
Mismatch Negativity (Fz)	−1.42	0.97	−1.26	1.34	−0.14
P3a	1.77	1.50	1.85	1.50	−0.05

Comparison of retested vs. not-retested subjects on baseline (Test Session 1) characteristics.

We hypothesized that the heritable neurophysiological and neurocognitive measures (e.g., [7], [22]) would show deficits in schizophrenia patients, exhibit at least moderate (ICCs>0.60) test-retest stability with little evidence of practice/maturation effects or relationships to fluctuations in clinical symptoms as is commonly assumed in the genetics of schizophrenia literature. We also hypothesized that the schizophrenia patients that failed to return for repeat testing would differ in their baseline (T1) characteristics with “non-returners” being generally more symptomatic, showing worse neurocognitive performance and having poorer functional status vs. those patients who returned for re-testing after 1 year (T2).

**Table 2:** One year stability of Clinical, Functional, Neurocognitive, and Neurophysiological Measures in Schizophrenia Patients and Nonpsychiatric Comparison Subjects.

	Schizophrenia Patients				Nonpsychiatric Subjects				Main Effects
	Time 1		Time 2		Time 1		Time 2		Group Time
	Mean	SD	Mean	SD	Mean	SD	Mean	SD	
**Clinical & Functional**									
SAPS Total	8.32	4.38	7.91	4.68					
SANS Total	13.60	4.55	13.97	4.57					
GAF-Modified	41.75	8.11	44.20	7.72					
SOF Total	47.57	6.27	47.78	6.54					
UPSA- Full	79.09	13.56	81.65	12.94					
UPSA-Brief	74.21	18.03	76.24	16.11					
**Neurocognitive**									
WRAT-3 Reading	43.01	7.95	44.42	6.88	51.31	4.53	51.55	4.85	G
LNS-Forward	11.34	3.05	11.75	3.18	14.61	2.90	14.42	2.70	G
LNS-Reorder	7.27	2.79	7.61	2.66	10.86	2.68	11.12	2.63	G
WCST Persev Resp	22.71	17.77	21.93	17.11	10.46	9.32	9.15	9.22	G
CVLT-II List A	34.41	10.49	36.68	12.26	52.41	10.34	56.88	9.67	G,T
**Antisaccade**	0.54	0.25	0.57	0.27	0.76	0.24	0.80	0.22	G
**Startle & PPI**									
Startle Magnitude	72.29	56.22	80.41	47.71	74.26	39.99	76.84	46.06	
Habituation	56.60	29.48	46.19	37.39	54.63	27.96	62.74	22.05	
PPI 30	34.69	26.34	31.78	22.85	38.81	21.70	38.98	21.15	
PPI 60	47.35	24.86	45.14	25.99	52.42	34.14	58.48	22.98	G
PPI 120	62.57	25.12	62.62	27.59	65.87	21.82	66.67	25.38	
**P50 & N100**									
P50 S1 Amplitude	2.26	1.62	2.48	1.86	3.18	2.10	2.96	2.17	
P50 S2 Amplitude	1.27	1.13	1.25	1.09	1.55	1.26	1.68	1.35	
P50 S1–S2	1.00	1.25	1.23	1.31	1.63	1.61	1.29	1.47	
P50% Suppression	37.37	45.43	39.94	48.15	45.00	46.81	35.72	46.26	
N100 S1 Amplitude	−2.53	2.23	−2.48	2.38	−4.16	3.24	−3.43	3.08	G
N100 S2 Amplitude	−1.73	1.59	−1.35	1.34	−2.18	1.81	−1.99	1.69	G
N100 S1–S2	−0.78	1.85	−1.14	1.84	−1.98	2.92	−1.44	2.35	
N100% Suppression	–	–	36.94	50.70	39.47	48.87	33.20	60.05	
**Mismatch Negativity & P3a**									
Mismatch Negativity	−1.42	0.97	−1.35	1.27	−2.56	1.60	−2.40	1.65	G
P3a	1.77	1.50	1.66	1.26	3.17	1.38	3.05	1.59	G

G: Significant group main effect.

T: Significant Time effect.

No Group by Time interactions were present.

## Methods

### Subjects

This study was approved by the University of California, San Diego Human Research Protections Program Institutional Review Board. All participants were assessed and judged to be capable of providing informed consent and, after subjects were given a detailed description of study procedures, written consent was obtained per UCSD IRB-approved protocols (IRB# 071128 and 071831) prior to each testing session. Participants included 546 subjects: 341 schizophrenia patients and 205 nonpsychiatric comparison subjects (NCS). Schizophrenia patients were recruited from community residential facilities and via physician referral. Normal comparison subjects were recruited through newspaper and internet advertisements and fliers posted at the UCSD Medical Center. All subjects received a urine toxicology screen to rule out recent drug use. In addition, patients and NCS were assessed using the Structured Clinical Interview for DSM-IV [26]. This interview was used to ensure that NCS did not meet criteria for an Axis I or Axis II Cluster A diagnosis Family history of psychiatric disorders was also assessed to ensure that NCS did not have a first-degree relative with a psychotic disorder [27]. In addition, patients did not have a current Axis I diagnosis other than schizophrenia. All subjects were carefully screened to ensure they had never experienced a neurologic insult, such as significant head trauma and/or loss of consciousness as per our established methods [24], [28], [29]. Audiometric testing was used to ensure that all participants could detect 40-dB tones at 1000 Hz for their data to be used in PPI, MMN, P3a, P50/N100 measures. There were neither statistically significant differences in hearing thresholds between the schizophrenia patient and NCS groups nor significant correlations between hearing thresholds and dependent measures. Data from subsets of these 546 participants who completed baseline testing were reported previously [29]–[32], including preliminary genetic association findings of a subset of 219 subjects of European ancestry and 76 subjects of African ancestry recently published in this journal [3].

**Figure 4:**
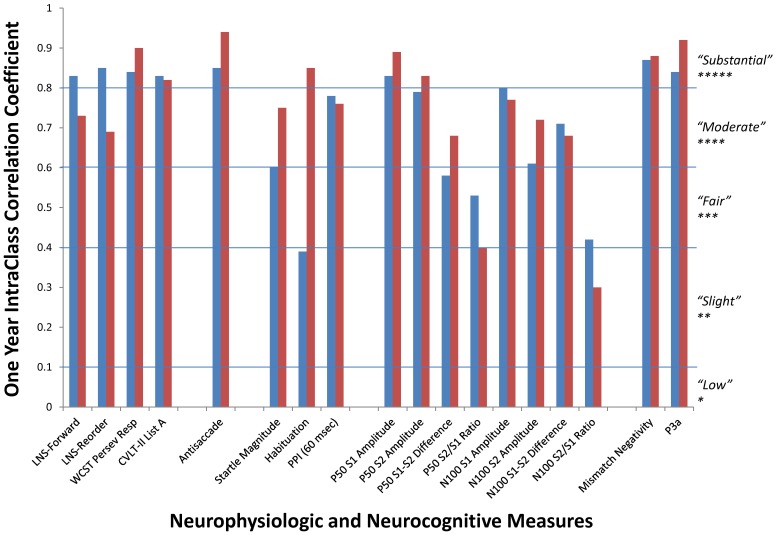
One year stability of candidate neurocognitive and neurophysiological endophenotypes. Intraclass correlation coefficients are shown for schizophrenia patients (blue; n = 163) and nonpsychiatric comparison subjects (red, n = 58). The mean retest interval was 364.57 (SD: 23.83) days.

**Figure 5:**
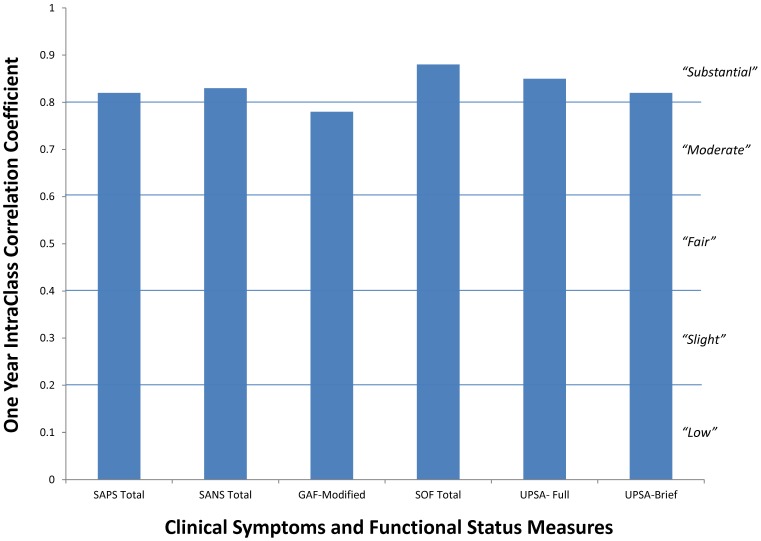
One year stability of clinical and functional measures in schizophrenia patients.

Schizophrenia patients (n = 163) and NCS (n = 58) were retested using identical procedures and the same fixed order of tests after approximately 1 year (mean number of days (SD) SZ: 365.34 (26.44); NCS: 363.95 (18.13) days; t = −0.37, p = 0.71) in order to characterize the stability of the measures as well as relationships to changes in clinical symptoms. As shown in Figure 1, of the 178 patients who were not retested, reasons for not returning were as follows: 62% were unable to be re-contacted, 26% were not invited to return by the study staff and investigators (e.g., unable to tolerate testing procedures, removed electrodes and/or showed excessive artifact during testing), 9% declined to return, and 3 subjects were excluded on retest (positive toxicology screen, failed hearing test). Medications were not experimentally controlled in this study. Among the retested patients, 6 were not treated with an antipsychotic (AP), 14 were prescribed 1^st^ generation AP, 113 patients 2^nd^ generation AP, and 30 received a combination of 1^st^ and 2^nd^ Generation AP when they came in for their initial (T1) test session. Over the retest interval, 44 patients underwent a change in the AP, with the majority (n = 22) switching from one to another primary 2^nd^ generation AP.

**Figure 6:**
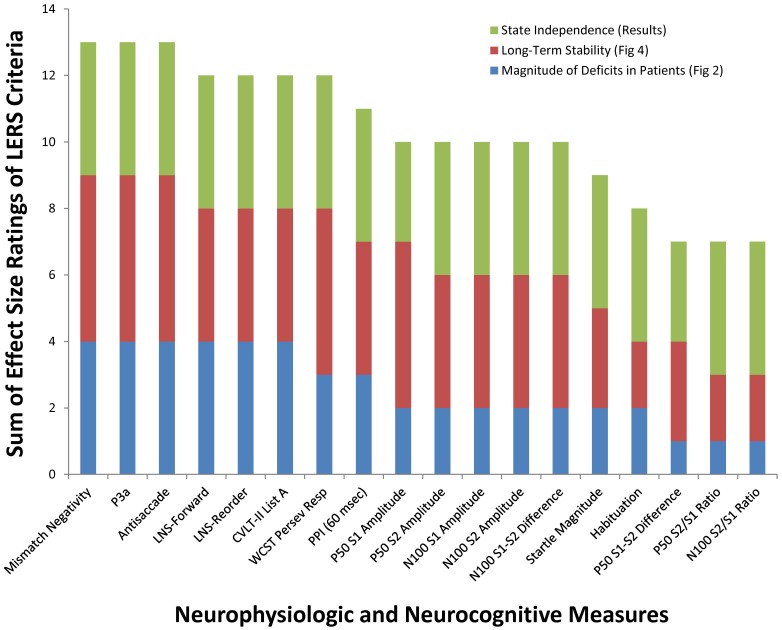
Summary of results: Longitudinal Endophenotype Ranking System LERS): Ranking biomarkers for use as endophenotypes in genomic studies and as biomarkers in clinical research studies. Neurophysiologic and neurocognitive measures are ranked based on the observed magnitude of deficits (1–4), test-retest reliability (1–5), and state independence (1–4) as shown in Figures 2, 3, 4, 5 and described in the results.

### Clinical and Functional Assessment Measures

In the schizophrenia patients, clinical symptoms were assessed with the Scale for the Assessment of Negative Symptoms (SANS; [33]) and the Scale for the Assessment of Positive Symptoms (SAPS; [34]). Functional *status* was assessed using a modified version of the Global Assessment of Functioning Scale (GAF; [35]) and the Scale of Functioning (SOF; [36]). Functional *capacity* was assessed using the UCSD Performance Based Skills Assessment (UPSA; [37]). To evaluate the relative stability of the abbreviated version of the UPSA, UPSA-Brief scores were also derived from the full scores in accordance with established methods [38].

### Neurophysiological and Neurocognitive Measures

The following neurophysiological and neurocognitive measures were assessed using our established parameters in a fixed battery: PPI [29], [30], startle habituation [39], P50 and N100 amplitudes and suppression measures [30], [40], MMN and P3a amplitudes [24], [31], [32], [41], [42], oculomotor antisaccade [43], simple auditory attention (Letter-Number Span Forward; LNS-Forward; [44], working memory (Letter-Number Span Re-order; LNS-Reorder; [44], [45]), immediate verbal recall (California Verbal Learning Test-2, Standard Form; CVLT-II; [46]), perseverative thinking and rule learning in response to verbal feedback (Wisconsin Card Sorting Test-64; WCST-64 [32]). The test order was as follows: PPI and startle habituation, oculomotor antisaccade, LNS-Forward, LNS-Reorder, CVLT-II, WCST-64 followed by a lunch break. After the break, particpants underwent EEG testing for P50 and N100 measures followed by MMN and P3a. For EEG testing, a 40 channel NeuroScan NuAmps system was used with sintered Ag/AgCl electrodes arranged in an electrode cap (EasyCap) with a forehead ground and nose reference. For N100, MMN, and P3a measures, amplitudes were measured relative to a 100 msec prestimulus baseline.

#### Mismatch negativity and P3a

Stimulation, recording, and analysis techniques for calculating MMN and P3a amplitudes followed our previously established methods [24], [28], [31], [32], [41], [42]. Subjects were presented with binaural stimulation (1 kHz computer-generated square wave stimuli, 85 dB[A] SPL, 1 msec rise/fall) with a fixed stimulus onset-to-onset asynchrony of 500 msec. Standard (P = 0.90; 50 msec duration) and deviant (P = 0.10; 100 msec duration) stimuli were presented in a pseudorandom order while participants watched a silent cartoon video. Signals were digitized at a rate of 1 kHz with system acquisition filter settings at 0.5–100 Hz. Testing was terminated after a minimum of 225 artifact-free responses to deviant stimuli was collected using the same automated procedures as described above. MMN and P3a waveforms were generated by subtracting ERPs in response to standard tones from the ERPs generated in response to the deviant tones. The MMN and P3a amplitudes were calculated from electrode Fz as the mean voltage from 135–205 and 250–300 msec ranges, respectively, consistent with established methods [24], [28], [32], [42].

#### Prepulse inhibition

Subjects were seated in a reclining chair in an upright position. The session began with a 5-min acclimation period with 70 dB[A] white noise that continued as the background throughout the session. All startle pulse stimuli were 40-msec 115 dB[A] bursts of white noise. Prepulse stimuli consisted of 20-msec noise bursts 15 dB above the 70 dB[A] background, presented 30, 60 or 120 msec prior to the onset of the startling stimulus. Our previous studies have demonstrated PPI in the 60 msec condition to be optimal for detecting deficits in schizophrenia patients [29], [30], [47], [48]. Five pulse-alone trials were presented at the beginning and end of the session to assess habituation. In each of Blocks 2–3, there were 8 pulse-alone and 8 of each of the three prepulse trial-types presented in a pseudorandom order with a 9–23 sec (15 sec average) intertrial interval.

#### P50 and N100 amplitudes and suppression

Subjects were tested a reclining chair to minimize myogenic artifacts. EEG data collection procedures, electrode locations, and data processing steps were performed following our established methods [24], [28], [29], [31], [32]. P50 and N100 processing was performed offline at electrode Cz [12], [49], [50]. Auditory click pairs (1 msec duration, 93 dB, 500 msec inter-click interval, 10 sec inter-pair interval) were presented to subjects. Testing was terminated after a minimum of 120 click pairs free of gross muscle or eye blink artifacts and were obtained using an automated threshold filter of +/−100 uVolts. Final data processing and EEG analyses were conducted offline and blind to group membership. Blink and baseline corrections were performed, followed by a second and fully automated artifact rejection screening for residual artifact exceeding +/−70 uVolts. Waveform averages were then generated and filtered using 24 dB/octave rolloff, (FIR) filters at 10–50 Hz for P50 and with a 30 Hz lowpass for N100 peak detections. P50 and N100 amplitudes in response to the first (S1) and second (S2) stimuli were selected automatically by a computer algorithm and subsequently reviewed offline by a research technician blind to subject diagnosis. For P50, the largest peak relative to a preceding negative trough in the 40–80 msec range was selected for S1 and S2 responses. S2 responses were further constrained to be within 10 msec of S1 responses [51]. N100 peaks relative to baseline were selected in the 65–135 msec range [40]. Individual P50 and N100 amplitudes in response to each of the clicks as well as the S1–S2 amplitude differences and percent suppression (1-[S2/S1]*100) were assessed.

#### Antisaccade (oculomotor inhibition)

Oculomotor recordings were obtained in a quiet, darkened room with subjects seated in front of a flat screen monitor with head stabilized using a chin rest or bite bar. Horizontal eye movements were measured using infra-red oculography (Eye Trak Model 310 eye movement monitor, Applied Science Laboratories, Waltham, MA). Stimuli consisted of square subtending about 0.35 degrees of visual angle. A stimulus was presented at central fixation, per our established methods [43], [51]. Following a random 2.4 to 3.6 sec interval, the fixation stimulus was turned off. Two hundred ms prior to fixation extinction, peripheral cues were illuminated at 10 or 15 degrees of visual angle to the right or left of center. After fixation extinction, this stimulus remained present for another 800 ms. Finally a 500 ms duration stimulus was presented indicating the location of a correctly performed antisaccade. Subjects were instructed to move their eyes as quickly and accurately as possible to the cue’s mirror image (same amplitude, opposite direction). Prior to commencing oculomotor testing, antisaccade cues were presented and subjects were required to point to, in addition to looking at, the proper location of gaze, insuring that all subjects understood task instructions. Antisaccade cues were yellow, all other stimuli were blue. Antisaccade data is analyzed with computerized pattern recognition software. After artifactual responses are removed the primary dependent measure is proportion of correct responses divided by the total number of (artifact-free) responses.

### Statistical Analyses

PASW Statistics version 18 was used for all statistical tests, with significance defined as p<0.01. This α-level reflects that there are 5 classes of endophenotypes under investigation (neurocognitive, antisaccade, startle/PPI, P50/N100, and MMN/P3a) and represents a reasonable balance of possible Type I and Type II errors for the large sample of subjects. Differential attrition was assessed by one-way Analyses of Variance (ANOVA) to determine if there were significant differences on baseline demographic, clinical, cognitive, and functional characteristics between those subjects who returned vs. failed to return for follow-up testing at one year. Subsequent analyses focused on the participants that returned for follow-up assessments. Effect-sizes (Cohen’s d) are presented for all group comparisons. Repeated measures ANOVA were performed with session and group, respectively, as within- and between-subjects factors. This analytic strategy enables the evaluation of whether: 1) schizophrenia patients differ from NCS on the key measures (i.e., a group main effect; effect size calculations shown in Figure 2); 2) the measures exhibit overall stability of means (i.e., a test session main effect; effect size calculations shown in Figure 3); and 3) the patients and NCS differ in the amount of change across the 1 year interval (group by test session interaction).

Stability was assessed via Type C intraclass correlation coefficients (ICC) using a consistency definition from a two-way mixed-effects model to provide an overall index of stability of T1 and second test (T2) measures in the patient and control groups separately. The ICC is a conservative estimate of test–retest reliability, because it is sensitive to group mean changes over time in addition to intra-subject variability. The following descriptors of reliability coefficients were used in accordance with established guidelines [52]: “Low” 0–0.1; “Slight” 0.11–0.40; “Fair” 0.41–0.60; “Moderate” 0.61–0.80; “Substantial” 0.81–1.0.

To determine whether changes in candidate endophenotypes were independent of fluctuations in clinical symptoms, T1–T2 change (Δ) values for endophenotypes and positive and negative symptoms over the retest interval were calculated. Pearson correlations were assessed between Δ values in endophenotype and SAPS and SANS.

## Results

### Evaluation of Differential Attrition

Since differential attrition can confound the interpretation of results, demographic, clinical, cognitive, and functional characteristics of schizophrenia patients at intake who returned (n = 163) vs. failed to return (n = 178) for follow-up testing were examined (α = 0.01; 80% power to detect d = 0.35 effect) prior to conducting primary analyses. Although this study aimed to retest only 100 SZ and 50 NCS after 1 year, efforts were made to retest as many SZ patients as possible. We anticipated that there may be some important pre-existing differences in the baseline characteristics of retested vs. non-retested patients that could account for the attrition rate and potentially compromise the validity of the retest results. Across 33 variables assessed (see Table 1), no significant differences were observed between these two groups of patients in demographic characteristics, global symptom ratings, functional scales, cognitive, or neurophysiological variables. Although there were no differences on global positive or negative symptom summary ratings in the schizophrenia patients, inspection of the 9 individual SAPS and SANS symptom ratings revealed that retested patients had slightly less severe ratings of delusions (means (SD) retested = 3.05, SD = 1.78; non-retested = 3.63 (1.51); F = 10.68, df = 1,338, p<0.001; d = 0.35). The groups did not differ in their level of independence in their community living situation, the distribution of living environments (i.e., board and care facilities, assisted independent living programs, independent living), SOF scores, GAF ratings, UPSA total scores, neurocognitive, or neurophysiological performances. Exploratory analyses of baseline demographic, cognitive, and neurophysiological variables in the NCS group revealed that the retested NCS were slightly older than the non-retested subjects (means (SD) retested = 45.97, SD = 10.71; non-retested = 40.13 (11.53); F = 11.4, df = 1,204, p<0.001; d = 0.50), but no other significant differences were observed across 24 statistical comparisons in this group. Thus, attrition does not appear to be substantially associated with key dependent variables for either the schizophrenia patients or the NCS.

### Group Differences and Evaluation of Potential Practice/Interval Effects

Significant deficits were present in the schizophrenia patients across all of the neurocognitive tests, antisaccade, PPI, N100 amplitudes, MMN, and P3a (Figure 2). Consistent with previous findings, no significant group differences were present on startle magnitude or habituation, and PPI in the 30 and 120 msec conditions [29]. In contrast to our expectations, significant deficits were not detected for any P50 measures, or N100 S1–S2 amplitude difference or suppression variables. Marginally significant test session effects were present on the CVLT-II test of immediate verbal recall with both groups showing improvements in their T2 assessments (Table 2, Figure 3). This improvement roughly equated to recalling an additional 1–4 words (out of 80 possible) over the retest interval. While no significant group by test session interactions were present across measures, only the CVLT-II approached significance (F_1,220_ = 5.21, p = 0.023).

### Evaluation of One-Year Test-Retest Stability

The majority of neurocognitive and neurophysiological measures demonstrated moderate to substantial one-year stability in both NCS and schizophrenia patients (Figure 4) with average ICCs across all measures exceeding 0.75. Indeed, 37 of 42 reliability assessments in the present study exceeded 0.60. Consistent with previous studies, P50 and N100 ratio measures did not show high stability. Similarly, percent startle habituation was not stable in the schizophrenia patients. The clinical and functional characteristics (Figure 5) of the schizophrenia patients also demonstrated substantial stability over the one year retest interval (mean ICCs = 0.83).

### Assessment of State Independence of Endophenotypes

As shown in Table 2, the schizophrenia patients exhibited significant clinical symptoms and functional impairments. Positive and negative clinical symptoms and functional assessment ratings were consistent across the 1 year retest interval. T1–T2 Δ scores in clinical symptoms (SAPS Δ Mean: 0.43, SD: 3.58; SANS Δ Mean = −0.45, SD = 3.53) and individual endophenotypes were calculated in order to assess the extent to which variation in clinical symptoms were associated with changes in endophenotypes across the retest interval. Changes in P3a were modestly associated with changes in positive (r = 0.21, p = 0.036) and negative (r = 0.26, p = 0.007) symptoms. Changes in negative symptoms were associated with changes in P50 S1 amplitude (r = 0.19, p = 0.045) and P50 S1–S2 difference (r = 0.21, p = 0.03). None of the remaining neurocognitive or neurophysiological measures were significantly associated with changes in positive or negative symptoms (all r<0.12, all p>0.10).

## Discussion

The majority of neurophysiological and neurocognitive measures examined in this study fulfill the criteria as valid endophenotypes for genomic studies and as robust biomarkers in clinical studies. Specifically, these candidate endophenotypes are: 1) associated with illness as they exhibit significant deficits in schizophrenia patients); 2) stable over 1 year in both patients and controls; 3) relatively insensitive to modest fluctuations in clinical symptoms; and 4) suitable for use as repeated measures since they do not show practice effects. Figure 6 summarizes the cumulative effect size ratings across of these 4 criteria, via a Longitudinal Endophenotype Ranking System (LERS; cf. [53]). Related studies have confirmed that many of these measures show significant deficits in clinically unaffected relatives of schizophrenia patients, are heritable and informative for the identification of genetic variation in schizophrenia [3], [7], [22], [54]. In addition, each measure has independently been the focus of intense scientific inquiry that has, in some cases, produced a rich literature describing informative animal models with predictive and construct validity [55] and detailed underlying neural and molecular substrates [3], [56].

### Deficits in Patients

Mismatch negativity, P3a, PPI, oculomotor antisaccade, and neurocognitive measures demonstrated significant deficits in schizophrenia patients and stability over the retest interval. A few of the neurophysiological measures are known to be sensitive to antipsychotic medication effects, which may have contributed to the results. Specifically, deficits in PPI and P50 suppression measures are known to be opposed by second generation antipsychotic medications [29], [50], [57]–[59]. Since this was a naturalistic study designed to validate the use of these measures in even larger-scale genomic studies where medications are virtually never experimentally controlled, subjects were not stratified on the basis of their medication type, gender, or smoking status–all factors known to influence PPI, P50, and other measures [29], [60]–[63]. It is therefore possible that these or other confounding factors may have contributed to the failure to detect significant and reliable P50 and N100 amplitudes and gating deficits in the schizophrenia patients. The modest reliability of ratio-based measures of P50 and N100 gating is consistent with previous studies of normal subjects over relatively brief retest intervals [64]–[68]. The results of this study support examining constituent S1 and/or S2 component amplitudes or non-ratio S1–S2 ERP amplitude difference measures. The Consortium on the Genetics of Schizophrenia (COGS) study which utilized a separate family-based sample of subjects also found that P50 and N100 individual response amplitudes and S1–S2 difference scores performed substantially better than gating ratios in heritability [7], [40] and genomic analyses [3].

### Stability Over 1-year

The stability of many of the neurophysiologic and neurocognitive measures in schizophrenia patients was somewhat higher-than-expected given the naturalistic study design that allowed for changes in medications and clinical status over the 1 year follow-up period. For example, 27% of patients underwent an addition, subtraction, or substitution of their primary antipsychotic medication (without considering the many dosage or adjunctive medication changes) over the test-retest interval. This robust stability raises the question of whether participants in this study were higher-functioning, asymptomatic, and therefore not representative of a “real world” community sample. An answer to this key question resides in the patient data: the retested vs. non-retested patients did not differ substantially in baseline characteristics. Many of the re-tested patients were neither high functioning nor asymptomatic (see Table 1). For example, 41% of the retested patients received maximal clinical severity ratings on hallucination items from the SAPS, 70% received maximum anhedonia and avolition ratings and, in terms of real-world function, 52% of patients required assistance with basic financial management.

With respect to the stability of clinical assessment measures, functional status and capacity are becoming more widespread in schizophrenia research. Following the MATRICS initiative [69]–[71], these types of assessments have been proposed as co-primary endpoints in clinical trial studies of potential cognitive-enhancing interventions. This study significantly advances the growing literature on the psychometric properties of functional outcome measures, including their long-term stability. For example, the stability of the Scale of Functioning (SOF) has not been reported previously. The substantial stability of the SOF (ICC = 0.88) supports its use in studies designed to characterize the global psychosocial and community functioning (e.g., [24], [28]. This scale provides important information about community functioning milestones such as occupational status, social relationships, and level of independence in living situations that can be targeted by novel pharmacologic and nonpharmacologic interventions [72]–[74]. Likewise, the finding of high 1-year stability of the UPSA and the abbreviated UPSA-Brief is consistent with and replicates recent reports over both short [75] and longer [76] retest intervals.

It is possible that the assessment of patients across more dynamic phases of illness, such as during the conversion from prodromal states to first episode schizophrenia [77]–[80] or during clinical exacerbations in already diagnosed schizophrenia patients might yield stability coefficients that were lower than what was observed in this more chronic outpatient cohort. Indeed, one could argue that the high stability observed in chronic patients is attributable to the fact that many of the patients have reached the nadir of their illness and are no longer experiencing dramatic fluctuations in the underlying neural networks that contribute to clinical symptoms and their related neurophysiological and neurocognitive function. It therefore remains an open question as to which if any of these measures will also serve as robust vulnerability markers in high risk populations [80], [81].

### Independent of Fluctuations in Clinical Symptoms

Neurophysiological and neurocognitive measures were relatively insensitive, accounting for <5% of the variance, to modest fluctuations in in clinical symptoms. The one exception was with the P3a event-related potential where changes in both positive and negative symptoms were correlated with changes in amplitude over the test-retest interval, consistent with previous findings [23], [82]. The absence of associations with symptom changes does not, however, invalidate the use of these measures as biomarkers of clinical, cognitive, or functional response to therapeutic interventions. Medications were not systematically controlled in this study, and thus changes in clinical symptoms were not necessarily ones that would optimally be “biomarker-sensitive” or indicative of a therapeutic drug effect. Many of the characteristics of the present measures such as deficits in patients, stability, utility as a repeated measure, are also criteria for biomarkers of response to experimentally controlled pharmacologic and non-pharmacologic interventions [16]. In fact, the Cognitive Neuroscience Treatment Research to Improve Cognition in Schizophrenia (CNTRICS) expert consensus panel has determined that only 2 neurophysiological measures–MMN and PPI– are “already mature” and among the most promising biomarkers for use in multi-site clinical trials [83].

In conclusion, the cumulative pattern of results suggests that these widely used neurophysiological and neurocognitive biomarkers are robust, reliable, state-independent, and therefore valid endophenotypes for ongoing genomic research studies where endophenotypes are used to fill the “gene-to-phene” knowledge gap. Genomic analyses from a subset of these participants have been recently reported in this journal [3]. Family-based genetic associations using these measures have also been reported for a separate sample from the multi-site COGS study [2]. Future studies are needed to assess the utility of these measures for predicting conversion to psychosis, as biomarkers of response to pharmacologic and nonpharmacologic [74], [84] treatments, for tracking disease progression across the course of illness [80], and for the delineation of schizophrenia-related abnormalities across genomic and neural networks [2], [3].
